# Preclinical Evaluation of Bioactive Small Intestinal Submucosa-PMMA Bone Cement for Vertebral Augmentation

**DOI:** 10.1021/acsbiomaterials.3c01629

**Published:** 2024-03-13

**Authors:** Chi Zhang, Xiongxiong Cai, Mei Li, Jing Peng, Jin Mei, Fangfang Wang, Rui Zhang, Yingjie Zhou, Shuyu Fang, Dongdong Xia, Jiyuan Zhao

**Affiliations:** † Department of Orthopaedic Surgery, The First Affiliated Hospital of Ningbo University, Ningbo University, Ningbo 315010, China; ‡ Zhejiang Key Laboratory of Pathophysiology, School of Medicine, Ningbo University, Ningbo 315211, China; § Key Laboratory of Precision Medicine for Atherosclerotic Diseases of Zhejiang Province, The First Affiliated Hospital of Ningbo University, Ningbo 315010, China; ∥ Institute of Biomaterials, The First Affiliated Hospital of Ningbo University, Ningbo 315010, China; ⊥ Department of Clinical Laboratory, The First Affiliated Hospital of Ningbo University, Ningbo 315010, China

**Keywords:** poly(methyl methacrylate) bone cement, small intestinal submucosa, vertebroplasty, biomechanics, biosafety, osteointegration

## Abstract

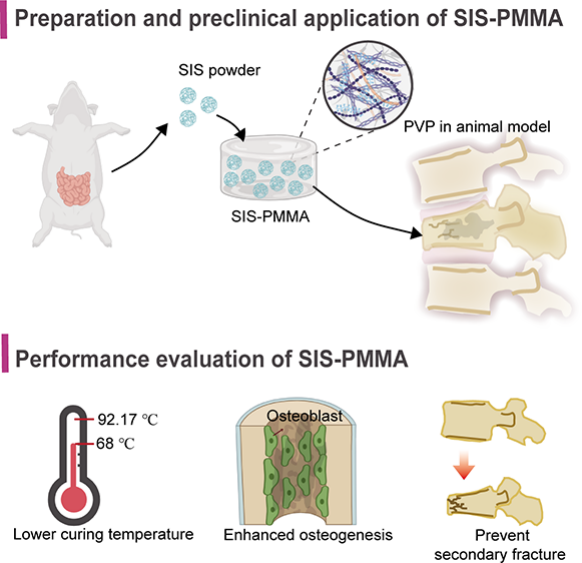

In vertebroplasty and kyphoplasty, bioinert poly(methyl methacrylate) (PMMA) bone cement is a conventional filler employed for quick stabilization of osteoporotic vertebral compression fractures (OVCFs). However, because of the poor osteointegration, excessive stiffness, and high curing temperature of PMMA, the implant loosens, the adjacent vertebrae refracture, and thermal necrosis of the surrounding tissue occurs frequently. This investigation addressed these issues by incorporating the small intestinal submucosa (SIS) into PMMA (SIS-PMMA). In vitro analyses revealed that this new SIS-PMMA bone cement had improved porous structure, as well as reduced compressive modulus and polymerization temperature compared with the original PMMA. Furthermore, the handling properties of SIS-PMMA bone cement were not significantly different from PMMA. The in vitro effect of PMMA and SIS-PMMA was investigated on MC3T3-E1 cells via the Transwell insert model to mimic the clinical condition or directly by culturing cells on the bone cement samples. The results indicated that SIS addition substantially enhanced the proliferation and osteogenic differentiation of MC3T3-E1 cells. Additionally, the bone cement’s biomechanical properties were also assessed in a decalcified goat vertebrae model with a compression fracture, which indicated the SIS-PMMA had markedly increased compressive strength than PMMA. Furthermore, it was proved that the novel bone cement had good biosafety and efficacy based on the International Standards and guidelines. After 12 weeks of implantation, SIS-PMMA indicated significantly more osteointegration and new bone formation ability than PMMA. In addition, vertebral bodies with cement were also extracted for the uniaxial compression test, and it was revealed that compared with the PMMA-implanted vertebrae, the SIS-PMMA-implanted vertebrae had greatly enhanced maximum strength. Overall, these findings indicate the potential of SIS to induce efficient fixation between the modified cement surface and the host bone, thereby providing evidence that the SIS-PMMA bone cement is a promising filler for clinical vertebral augmentation.

## 1 Introduction

With time, the world population is aging, and it has been estimated that by 2050, the number of osteoporosis patients will reach 1.55 billion.^1^ In elderly people, osteoporotic vertebral compression fractures (OVCFs) are the most frequent type of fracture and are a major health concern. Over the past few decades, one of the main treatments for osteoporotic lower back pain and OVCF in geriatric patients has been minimally invasive percutaneous vertebroplasty, including percutaneous vertebroplasty (PVP) and percutaneous balloon dilatation (PKP).^2,3^ These procedures provide a remarkable, short-term therapeutic effect. Poly(methyl methacrylate) (PMMA) is the most widely used vertebroplasty material because of its vast advantages, including good tissue bonding, high strength, and biosafety, which help fill and stabilize fractured vertebral bodies, thereby substantially relieving pain.^4^ However, clinically, PVP and PKP have multiple problems and risks, such as adjacent vertebrae refracture, pulmonary embolism, aseptic loosening and shifting, thermal necrosis, and neurological dysfunction. The literature indicated that these problems or risks are because of PMMA’s increased elastic modulus, high setting temperature, and lack of bone-bonding ability.^5−8^

Currently, the incorporation of inorganic fillers, including hydroxyapatite (HA),^9^ β-tricalcium phosphate (β-TCP),^10,11^ and bioglass,^12^ has proved to be an effective method to reduce the PMMA-associated risks. However, the application of these hybrid bone cements in spinal repair requires more research because of their poor anticollapse properties and injection performance. Furthermore, bioactive materials, such as linoleic acid,^13^ nano-MgO,^14^ growth factor,^15^ and biomolecule,^16^ can also be employed to improve the physical properties and biological performance of PMMA cement. It has been reported that after modification, the proliferation, adhesion, spread, and osteogenic gene expression of osteoblasts significantly improve. However, in some studies, biomechanical impairment may result from the direct incorporation of these substances into PMMA. Moreover, their safety and efficacy have not been validated in animal lumbar vertebral defect models. Therefore, for OVCF clinical treatment, the development of a modified PMMA-based bone cement with adapted mechanical properties and excellent biological activity in vitro and in vivo is very important and challenging.

Small intestinal submucosa (SIS) is a natural biomaterial that comprises a collagenous extracellular matrix with various factors, which is chemically similar to the natural bone matrix in composition and structure.^17^ It has been approved by the FDA, and the xenogenically derived SIS has been widely used in various tissue repair studies. Furthermore, in vivo has indicated that it is nonimmunogenic.^18−21^ Several studies have verified that SIS can provide a good microenvironment to facilitate the proliferation, attachment, and osteogenic differentiation of fibroblast bone, osteoblasts, and marrow mesenchymal stem cells.^17,22−25^ In our previous study, SIS was applied to modify commercially available PMMA cement to optimize the biological performance and in vivo repair effect of the basic material.^26^ Though PMMA with 20 or 40% SIS powder showed excellent bioactivity to enhance the new bone formation, the compressive strength of the constructs was hard to meet the requirement of clinical application (ISO5833), which was essential for clinical application. In another report, the researchers constructed a mineralized SIS acellular matrix-PMMA through the addition of phosphate and soluble calcium. The constructs were demonstrated to promote new bone formation.^27^ However, preclinical evaluation of such bioactive bone cements was never measured in previous studies.

PMMA-based bone cement as a product, the preclinical evaluation is a crucial step for clinical translation. Based on the previous studies, the present study mainly conducted a series of preclinical studies on the clinical application of the bioactive bone cement, analyzing and evaluating its clinical application value. Based on this purpose, we modified the size of SIS powder and adjusted the proportion of SIS addition to explore its relationship with the pore size and mechanical properties of bioactive bone cement. On this basis, preclinical evaluation has been conducted systematically. Polymerization temperature, handling time, biosafety, and larger animal model evaluation, which are essential for clinical application, have been evaluated in the present study. We found that the synthesized bioactive bone cement not only enhanced bone healing and regeneration but also showed the stability, operability, and biosafety required by clinical practice and avoided secondary fractures. Therefore, SIS-PMMA bone cement might be a promising candidate filler for clinical vertebral augmentation.

## 2 Materials and Methods

### 2.1 Ethical Statement

All procedures that were performed on animals followed Chinese legislation on the care and use of laboratory animals. This investigation was authorized by the Animal Care and Use Committee of Ningbo University (number: 12625).

### 2.2 SIS-PMMA Cement Preparation

The basic material utilized was PMMA bone cement (Mendec Spine, Tecres S.P.A., Verona, Italy) with powder formulation comprising PMMA polymer (67.5 wt %), barium sulfate (30.0 wt %), and benzoyl peroxide (2.5 wt %), whereas the liquid formulation included methyl methacrylate monomer (99.1 wt %), N, N-dimethyl-p-toluidine (0.9 wt %), and 75 ppm hydroquinone. For dried decellularized SIS preparation, the fresh small intestine was mechanically dissociated, defatted, decontaminated, digested enzymatically, and then lyophilized. This SIS was then pulverized with the help of TissueLyser-24 (Jingxin, Shanghai, China). To acquire powdered SIS, TissueLyser was utilized twice at 45 Hz for 30 s; the resulting powder was passed through a 50-, 100-, and then 200-mesh test sieve. The powder size distribution and morphology of the PMMA and SIS powders were assessed via scanning electron microscopy (SEM; Phenom Pro, The Netherlands).

**Figure 1:**
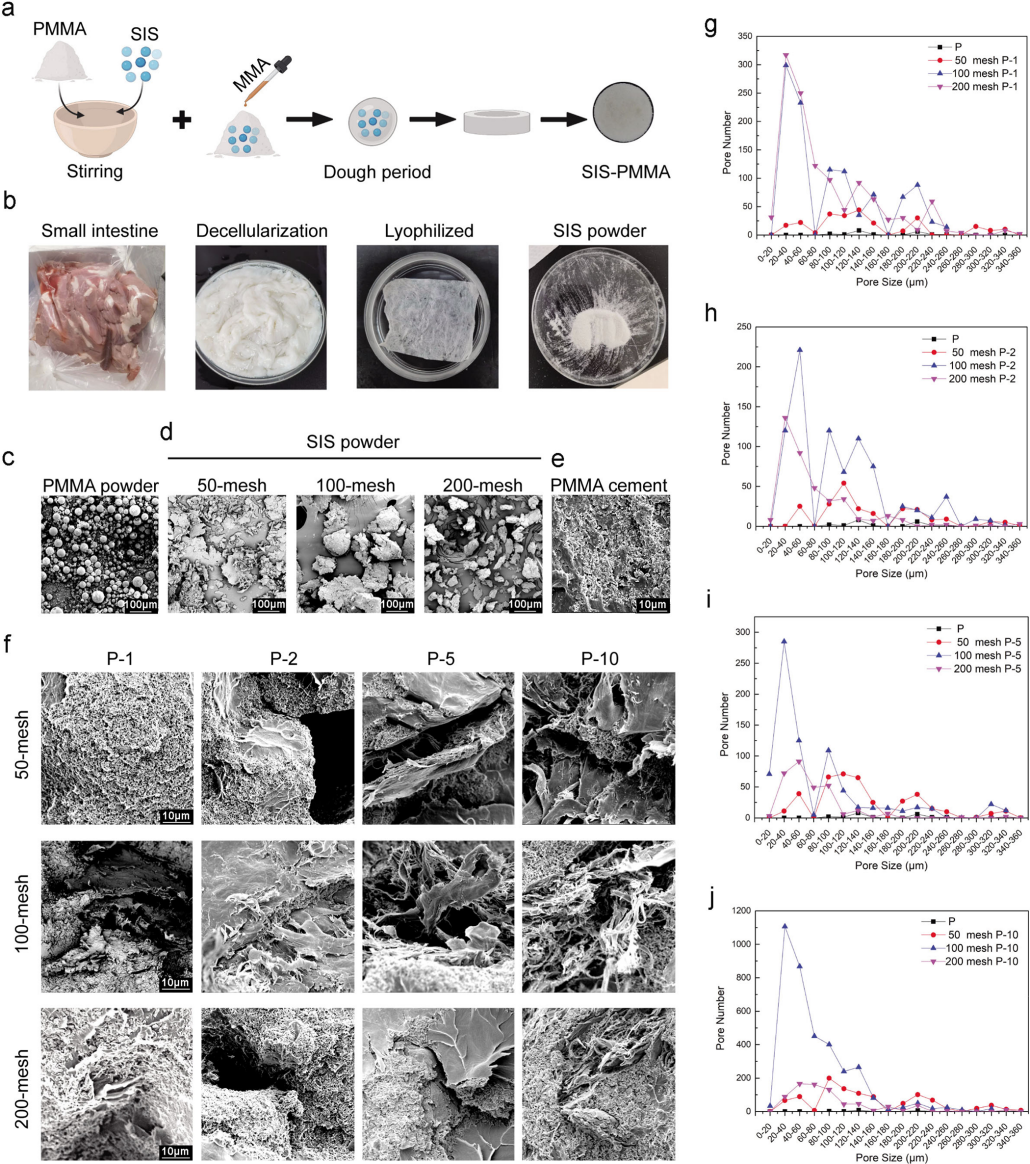
Synthesis and characterization of SIS-PMMA. (a) Schematic illustration of the SIS-PMMA bone cement preparation. (b) Fabrication of decellularized porcine SIS powder. SEM images of (c) PMMA and (d) SIS powder passed independently through a 50-, 100-, and 200-mesh test sieve. Scar bar, 100 μm. Cross-sectional SEM images of (e) PMMA bone cement and (f) PMMA bone cement with different proportions and powder sizes of SIS. Scar bar, 10 μm. (g–j) Pore number and pore size distribution of the SIS-PMMA bone cement.

The SIS-PMMA bone cement was prepared by mixing PMMA with SIS powder and then adding liquid MMA to make a usable dough for injection molding (Figure 1a). Five composite cements were prepared with different SIS contents, including PMMA + 1% SIS, PMMA + 2% SIS, PMMA + 5% SIS, PMMA + 10% SIS, and PMMA + 15% SIS. The volume of MMA used for the SIS-PMMA groups was according to the manufacturer’s suggestion for commercial PMMA. Table 1 indicates the compositions of the SIS-PMMA bone cements. Pure PMMA and five types of composite cement containing 1, 2, 5, 10, and 15 wt % SIS were designated P, P-1, P-2, P-5, P-10, and P-15, respectively.

**Table 1:** Composition of PMMA-Based Cement Containing SIS Powder

sample	powder (g) Mendec-S	powder (g) SIS	Mendec-L (mL/g)
P	1		
P-1	1	0.01	0.5
P-2	1	0.02	0.5
P-5	1	0.05	0.5
P-10	1	0.1	0.5
P-15	1	0.15	0.5

### 2.3 Cross-Section Morphology Observation and Pore Properties of SIS-PMMA Cement

Vise-snapped PMMA and SIS-PMMA (Φ10 mm × 2 mm, *n* = 3) were coated with platinum, and the inner microstructure was examined under SEM (Phenom Pro, Netherlands). Each sample’s pore number and size distribution were assessed using SEM images. ImageJ software was employed to measure 15 micrographs per group. The suitable proportion for subsequent tests was identified from these tests.

### 2.4 Biological Characterization

#### 2.4.1 Cell Culture and Cell Proliferation

MC3T3-E1 cells were obtained from the Type Culture Collection of the Chinese Academy of Sciences (Shanghai, China) and cultured in αMEM medium (Gibco, Carlsbad, CA, USA) augmented with fetal bovine serum (10%, Corning). PMMA and SIS-PMMA (PMMA + 2% SIS, PMMA + 5% SIS, PMMA + 10% SIS, and PMMA + 15% SIS) bone cement were prepared into disc-shaped specimens (10 mm diameter and 2 mm thickness). Using ultraviolet light and ozone, all the polymerized crude specimens were sterilized before the test and soaked in PBS for 1 week before cell culture.

To determine if the materials emitted any soluble toxic factors, the MC3T3-E1 cells were co-cultured with the specimens in a 12-well Transwell system (3.0-μm pore size; Corning). Briefly, 30,000 cells were propagated in the lower chambers, and in the upper chamber, 1 disc-shaped specimen was added. Subsequently, the optical density was assessed at 450 nm on days 1, 3, 5, 7, and 9 via Cell Counting Kit 8 (BS350B, Biosharp, China). Furthermore, 1 disc-shaped specimen was placed in the bottom of the 24-well plate, and approximately 30,000 cells were seeded onto its surface for 12 h to allow full cell adherence with the specimen. The next day, specimens were transferred to new wells and cultured in fresh medium (500 μL) at 37 °C in 5% CO_2_. Each group was assigned 3 parallel wells, and 4 samples were tested/group. For cell proliferation analysis, CCK-8 reagent (BS350B, Biosharp, China) was added to the culture medium on days 1, 3, 5, and 8. The absorbance (*A*) value at 450 nm was measured using an enzyme labeler, and the relative proliferation (%) was calculated using the following formulas:
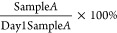
1

#### 2.4.2 Osteogenic Differentiation Assay

MC3T3-E1 cells (6 × 10^4^) were seeded onto PMMA and SIS-PMMA cements (Φ10 × 2 mm, *n* = 3). After 5 days, mineralized nodule deposition was assessed via Alizarin red S staining. Briefly, MC3T3-E1 cells were preserved in 4% paraformaldehyde and then dyed for 30 min with 2% alizarin red staining solution/PBS (G8550; Solarbio) at 37 °C. Then, a camera was utilized to record the stained Ca on the bone cement.

### 2.5 Physical Characterization of the SIS-PMMA Cements

#### 2.5.1 Mechanical Properties

The mechanical properties of PMMA and PMMA with 5, 10, and 15% SIS were assessed via a universal material testing machine (Shimadzu, Japan) based on ISO 5833. Furthermore, for compressive strength and modulus tests, cylindrical specimens (6 mm diameter and 12 mm height) were prepared by using a polytetrafluoroethylene (PTFE) mold and loaded to failure at 20 mm/min speed. Using the recorded stress–strain curves and the first-order derivative of the curves, the compressive strength and compressive modulus were assessed, respectively. Furthermore, flat plate specimens with 75 mm length, 10 mm width, and 3.3 mm thickness were also generated using PTFE mold to test the three-point flexural properties at a crosshead rate of 5 mm/min until the sample broke. To ensure measurement accuracy, the average value of five specimens of each SIS-PMMA ratio was obtained. Each specimen’s flexure strength and flexure modulus were assessed based on their expressions in ISO 5833–2002 and ISO 14125:1998.

For each test specimen, the flexure strength, *B*, in megapascals (MPa), from the expression:
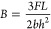
2

For each test specimen, calculate the flexure modulus, *E*, in megapascals (MPa), from the expression:
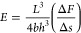
3where *F* is the force at break in newtons; Δ*s* is the difference in deflection between *s*″ and *s*′; Δ*F* is the difference in load *F*″ and load *F*′ at *s*′′ and *s*′, respectively; *b* is the average measured width of specimen, in millimeters; *h* is the average measured thickness of specimen, in millimeters; *L* is the span, in millimeters (64 mm).

#### 2.5.2 Temperature of the Polymerization Reaction

The weight of the PMMA powder added in PMMA, PMMA + 5%SIS (mass ratio of SIS:PMMA = 0.05:1), and PMMA + 10%SIS (mass ratio of SIS:PMMA = 0.1:1) groups was 2 g. After prepolymerized PMMA and SIS powders were thoroughly mixed, the liquid MMA monomer was added to initiate the MMA polymerization reaction (Figure 1a) by using a 5 mL syringe barrel as a reaction vessel. Immediately after mixing, the temperature was recorded for 20 min with the help of a type K thermocouple (Taishi Electronic Industry Co., Taiwan). The temperature variation curve was plotted with time as the horizontal axis and the real-time temperature as the vertical axis. These analyses were carried out at an ambient temperature of 23 ± 1 °C and not <40% relative humidity. For each group, the average value of five replicates was measured.

#### 2.5.3 Handling Times Tests

The surgeon’s handling time is important for the clinical application of the bone cements. The handling time comprises four phases: mixing, waiting, working, and hardening. Here, these four stages were evaluated at each SIS-PMMA ratio to investigate the influence of the SIS addition on the operation properties of the bone cement. The weight of the PMMA powder added in PMMA, PMMA + 5% SIS (mass ratio of SIS:PMMA = 0.05:1), and PMMA + 10% SIS (mass ratio of SIS:PMMA = 0.1:1) groups was 2 g. The same surgeon performed the test four times for each group.

### 2.6 Ex Vivo Augmentation of Goat Vertebrae

#### 2.6.1 Ex Vivo Decalcified Bone Model and Defect Creation

The ex vivo biomechanical properties of modified and pure PMMA employed in a vertebral augmentation procedure were elucidated. From a slaughterhouse, six goat vertebral lumbar sections (i.e., L2–L5) were obtained. Each vertebral section was carefully detached and cleaned from soft tissue, and the disc and transverse spinous sections were processed and preserved for 24 h in 10% formalin. The specimens were then washed with PBS and decalcified in 0.4916 mmol/L EDTA-Na2 with shaking for 6 days. The solution was replaced every day. All specimens were measured for bone mineral density (BMD, GE Lunar DPX Prodigy, Metriscan, USA) before and after decalcification to confirm osteoporosis. Subsequently, by covering the dental acrylic cement, both decalcified vertebral end plates were flattened. A compression fracture model was constructed by placing the specimens between two parallel plates of a mechanical tester (Shenzhen Sansi Testing Co., Ltd., China) and compressing at a 10 mm/min rate to 25% of the initial height.

#### 2.6.2 Cement Augmentation and Biomechanical Compression Test

Nine decalcified compression fracture vertebral bodies were randomly categorized into PMMA, PMMA + 5% SIS, and PMMA + 10% SIS bone cement groups. Then, the three types of cement were slowly injected until the defect was completely filled. After 24 h of bone cement injection, the height of each vertebra and the diameter of both ends of the covered bone cement were measured with vernier calipers for biomechanical measurements. Briefly, the cement specimen’s compressive strength and elastic modulus were elucidated by loading them to 25% of the initial height at 10 mm/min speed in a universal testing machine equipped with a 10 kN load cell. The stress–strain curves were utilized for elastic modulus and compressive strength assessment.

### 2.7 Biological Safety Evaluation of SIS-PMMA Cement In Vivo

#### 2.7.1 Preparation of Extracts of Cement Samples

Extracts of PMMA and SIS-PMMA bone cement were generated per ISO 16886.12:2017 (Biological evaluation of medical devices–Part 12: Sample preparation and reference materials). First, the surface and bottom of cured cement samples (3.3 mm thickness, 10 mm width, 75 mm length, *n* = 6/group) were sterilized by exposing them to ultraviolet light for 30 min. Then, the samples were incubated with either 10.9 mL of normal saline (Kelun Industry Group Co., Ltd., Sichuan, China) or olive oil (Yibin Guangquan Food Liability Co., Ltd., Sichuan, China) and shaken at 70 rpm and 37 °C. The specimen surface area/soaking solution ratio was 6 cm^2^/mL. After 1 week of soaking, the specimens were removed from the solution; the polar (in normal saline) and nonpolar (in olive oil) extracts were acquired and stored at 4 °C for biological safety analysis.

#### 2.7.2 Acute Systemic Toxicity Analysis in a Mouse Model

The acute systemic toxicity of PMMA and SIS-PMMA bone cement was assessed per ISO 16886–11:2011 (Biological evaluation of medical devices–Part 11: Tests for systemic toxicity). The ICR mice (weight = 27 to 35 g, *n* = 40, male) acquired from the SLAC Laboratory Animal Co. Ltd. (Zhejiang, China) were randomly grouped into blank control, PMMA, PMMA + 5% SIS, and PMMA + 15% SIS. Each group had an equal number of polar and nonpolar extract-injected mice. Olive oil and normal saline groups were set as the blank controls for nonpolar and polar extracts, respectively. The 50 mL/kg/day of polar and nonpolar extracts were injected intravenously and intraperitoneally, respectively. Each mouse’s body weight was recorded before and after the injection. Furthermore, clinical symptoms, including weight loss, prostration, convulsion, and death, were observed for 72 h postinjection (*n* = 5).

#### 2.7.3 Subacute Systemic Toxicity Analysis in a Mouse Model

The subacute systemic toxicity of PMMA and SIS-PMMA bone cement also followed the guidelines of ISO 16886-11:2011. Here, ICR mice (weights = 27 to 35 g, *n* = 48, male) were randomly categorized into the experimental and blank control groups. Each group had an equal number of polar and nonpolar extract-injected mice. Polar extracts (10 mL/kg) and normal saline (negative control) were intravenously administered daily for 7 days. All mice were fed for 14 days until the end point of this analysis. Each group’s general conditions and toxic reactions were observed daily, and at the end of the test, blood was taken from the eye socket for hematological and blood biochemical indicators analyses (*n* = 6), conducted via an automated biochemical analyzer (Hitachi 7600-110, Japan) and an automated hematology analyzer (Mindray, BC-2800vet, China). The hematological indicators included neutrophil (NE), white blood cell (WBC), eosinophilic granulocyte (EO), monocyte (MO), lymphocyte (LY), and basophilic granulocyte (BA) counts as well as hemoglobin (Hb) and mean corpuscular hemoglobin (MCH) concentrations, platelet (PLT) count, mean corpuscular volume (MCV), hematocrit (HCT), red blood cell (RBC) count, and red cell distribution width (RDW), whereas the blood biochemical indicators included alanine aminotransferase (ALT), chlorine (Cl), total protein (TP), albumin (ALB), aspartate aminotransferase (AST), blood glucose (GLU), creatinine (CREA), potassium (K), direct bilirubin (DBIL), alkaline phosphatase (ALP), sodium (Na), blood urea nitrogen (BUN), and calcium (Ca) levels.

### 2.8 In Vivo Animal Experiment

#### 2.8.1 Surgical Procedures

In this investigation, 12 mature New Zealand rabbits (male, weigh = 2.5–3.0 kg) were purchased from Charles River Laboratories (Shanghai Slac Laboratory Animal Co., Ltd., China) and categorized into PMMA, PMMA + 5% SIS, and PMMA + 10% SIS groups (*n* = 4). After 1 week of acclimatization, pentobarbital (3%, 1 mL/kg) was injected into the rabbit’s ear vein while in the prone position and sterilized anesthetization. The injection points were selected by the vertebral mastoid location according to reported methods.^28^ By palpation, the L5 and L6 spinous processes were confirmed, and then a 14G bone marrow cannula (Ningbo HICREN Biotechnology Co., Ltd.) was administered to the bone point at 8 mm. A syringe attached to a 1.0 mL diameter needle was used to inject three biomaterials (pure PMMA, PMMA + 5% SIS, and PMMA + 10% SIS) into the corresponding positions. The subcutaneous tissue and skin were sutured via an absorbable suture (Jinhuan Corp, Shanghai, China). After the surgery, rabbits were intramuscularly injected with penicillin (200,000 U, Shengwang Corp, Shandong, China) three times; immediately, 24 and 48 h postsurgery. The animal’s mental state, wound healing, activity, and diet consumption were monitored postoperatively.

#### 2.8.2 Microcomputed Tomography (micro-CT) Evaluation

After 12 weeks of the surgery, rabbits were euthanized, their lumbar vertebra (*n* = 8/group) was excised, and the soft tissues were removed before soaking in neutral formalin. A micro-CT scan system (NEMO Micro-CT; PINGSENG Healthcare, China) was employed to examine all the specimens, and their image was taken at 50 mm resolution (using 0.55 mA and 70 kV). For the three-dimensional (3D) reconstruction of the images, computer software (Recon; PINGSENG, Shanghai, People’s Republic of China) was used. Furthermore, with the help of the avatar software, the regenerated bone volume fraction (bone volume/tissue volume; BV/TV) was assessed.

#### 2.8.3 Hard Tissue Section

All vertebral specimens were fixed in formaldehyde–acetic acid–ethanol fixative solution for 48 h, dehydrated in successive alcohol concentrations (60% to absolute), and then cleared with xylene before being embedded in PMMA under vacuum for 5 h and polymerized in a 37 °C water bath. After the samples hardened, their 10 μm sections were cut via a diamond tissue microtome (SAT-001-E300CP; EXAKT, Hamburg, Germany) and then dyed with Van Gieson and TRAP stains.

#### 2.8.4 Organ Cytotoxicity

After 12 weeks postsurgery, the heart, spleen, liver, kidney, and lung were dissected and preserved in neutral formalin (Solarbio Life Science, Beijing, China), subjected to gradient dehydration, then dipped in paraffin, and cut into 5 μm slices. The samples were stained with hematoxylin-eosin (H&E), and an Eclipse 80i microscope (Nikon, Japan) was employed for histological analysis.

#### 2.8.5 Biomechanical Test

The rabbits were euthanized 12 weeks after PVP, and compression tests were carried out to investigate the compressive strength and compressive modulus of the experimental vertebra. Briefly, the lumbar vertebra with PMMA, PMMA + 5% SIS, and PMMA + 10% SIS bone cement were dissected (3 samples/group). Cap-like bone cement-covered samples were utilized for assessing the strength under compression via a mechanical tester with a displacement velocity of 20 mm/min. The corresponding stress and strain data were recorded.

### 2.9 Statistical Analysis

For all statistical analyses, SPSS software (Chicago, IL) was used, and the quantitative data are presented as the means ± standard deviations (SD). Statistical significance in multiple comparisons was assessed via one-way analysis of variance (ANOVA) followed by a post hoc test (Bonferroni). *p* < 0.05 was set as the threshold value indicating a statistically significant difference; *p < 0.05, **p < 0.01, ***p < 0.001.

## 3 Results

### 3.1 SIS-PMMA Cement Composition and Properties

The SIS-PMMA bone cement was prepared by mixing PMMA with SIS powder and then polymerizing it with the liquid MMA monomer (Figure 1a). The decellularized SIS powder was obtained from the pig intestine with different sizes through 50-, 100-, and 200-mesh test sieves by following the process in Figure 1b. The powder morphologies of the PMMA and SIS observed by SEM are shown in Figure 1c,d. The SIS powder of the 100- and 200-mesh groups indicated an obvious particle morphology and relatively uniform particle size, with larger average diameters of the 100-mesh group (123.3 ± 33.57 μm) than the 200-mesh group (52.4 ± 18.58 μm). Furthermore, the cross-sectional SEM images of the cured PMMA and SIS-PMMA bone cement (Figure 1e,f) revealed that PMMA had a dense cross-section, while SIS-PMMA (with varying SIS particle sizes and SIS:PMMA ratios) had a random distribution of fibrous SIS and interconnected pores. ImageJ software was employed to assess the pore number and size distribution of the groups (Figure 1g–j). In comparison to the PMMA group, the composite groups exhibited increased pore numbers. Moreover, 100-mesh test sieve SIS powder, with 2, 5, and 10% ratios in cement, had significantly more pores relative to that of the 50- and 200-mesh groups, which is adequate for cell migration. Specifically, the PMMA + 10% SIS composite group through a 100-mesh test sieve had an elevated pore number compared to other groups, covering a wide pore size distribution (10–160 μm).

### 3.2 Cell Viability and Osteogenic Differentiation

**Figure 2:**
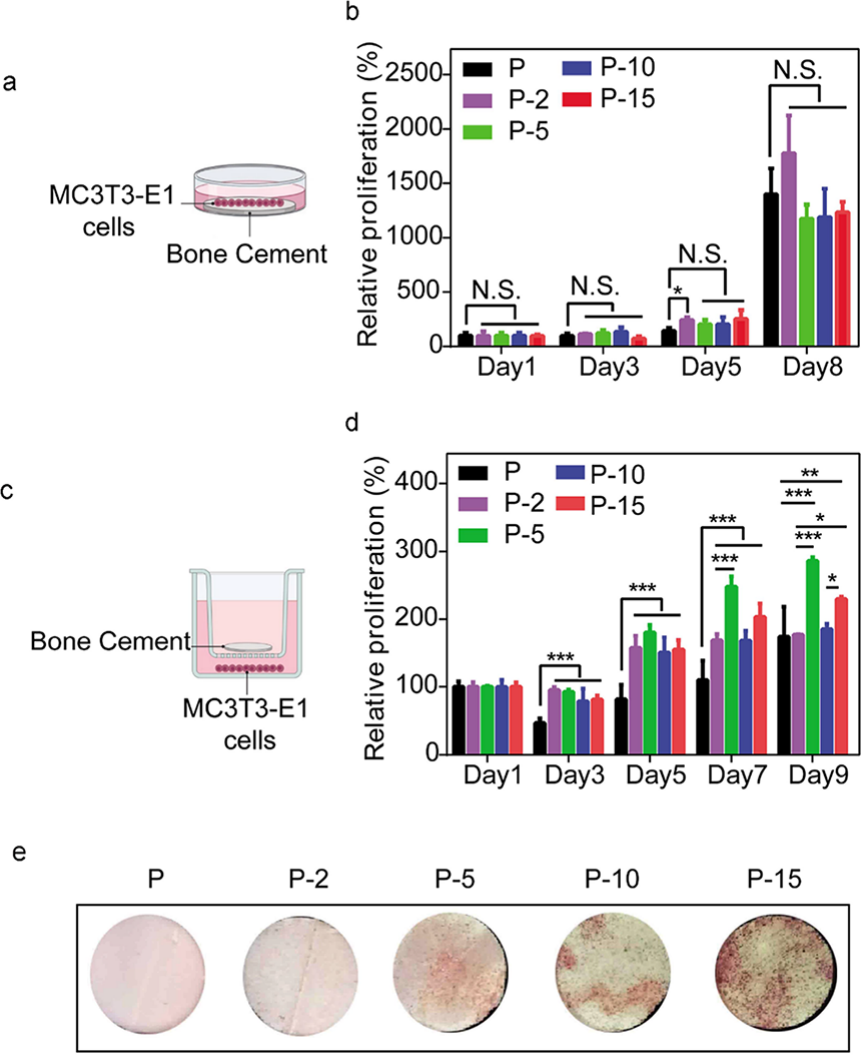
Cytocompatibility and osteoinductive property of the PMMA and SIS-PMMA bone cement. (a, b) Relative proliferation rate of MC3T3-E1 cells cultured on PMMA and SIS-PMMA bone cement was assessed on days 1, 3, 5, and 8 via CCK-8 assay. (c) Using an in vitro Transwell insert model, (d) relative proliferation rate of MC3T3-E1 cells incubated with PMMA and SIS-PMMA was assessed on days 1, 3, 5, 7, and 9. (e) Alizarin Red staining of mineralized calcium nodule-formed osteoblasts cultured on SIS-PMMA for 5 days. *, *p* < 0.05, **, *p* < 0.01, and ***, *p* < 0.001. The error bars indicate standard deviations.

To determine the in vitro effect of SIS-PMMA bone cement, MC3T3-E1 cells were seeded on PMMA and SIS-PMMA cement (Figure 2a). The cell proliferation on the SIS-PMMA at days 1, 3, and 8 was not significantly different from that on the PMMA bone cement regardless of the SIS ratio (Figure 2b). On day 5, the cell growth on the P-2 bone cement was higher than that observed on the PMMA bone cement, while that on the P-5, P-10, and P-15 cements did not significantly differ from that on PMMA. This indicates that there was no significant variability in cell safety between SIS-PMMA and commercial PMMA cement. Compared with commercial PMMA, the composite cement could also serve as a platform for the attachment and proliferation of cells.

Furthermore, MC3T3-E1 cells were exposed to PMMA and composite cement via a Transwell insert model (Figure 2c). After 3 days of culture, the CCK-8 test indicated a substantially increased relative cell proliferation on SIS-PMMA (P-2, P-5, P-10, P-15) than on PMMA at days 3, 5, and 7 (Figure 2d). Moreover, on day 9, the cell growth on P-5 and P-15 bone cement was more than that on the PMMA bone cement, with no marked difference between P-2 and P-10 cements compared with PMMA. The results indicated that SIS-PMMA induced cell proliferation to a greater extent than PMMA. The literature indicates that SIS has no toxic effects. The bone cement toxicity is primarily due to its components and unpolymerized monomers. SIS addition did not increase the toxicity of PMMA, indicating that SIS had no significant effect on PMMA polymerization. Additionally, the osteogenic differentiation of MC3T3-E1 cells cultured on the composites was also evaluated (Figure 2e). On day 5, a representative sample of abundant mineralized nodules was observed in the composite group, which revealed an increase in the mineralized nodules with increasing SIS levels.

### 3.3 Mechanical Properties

**Figure 3:**
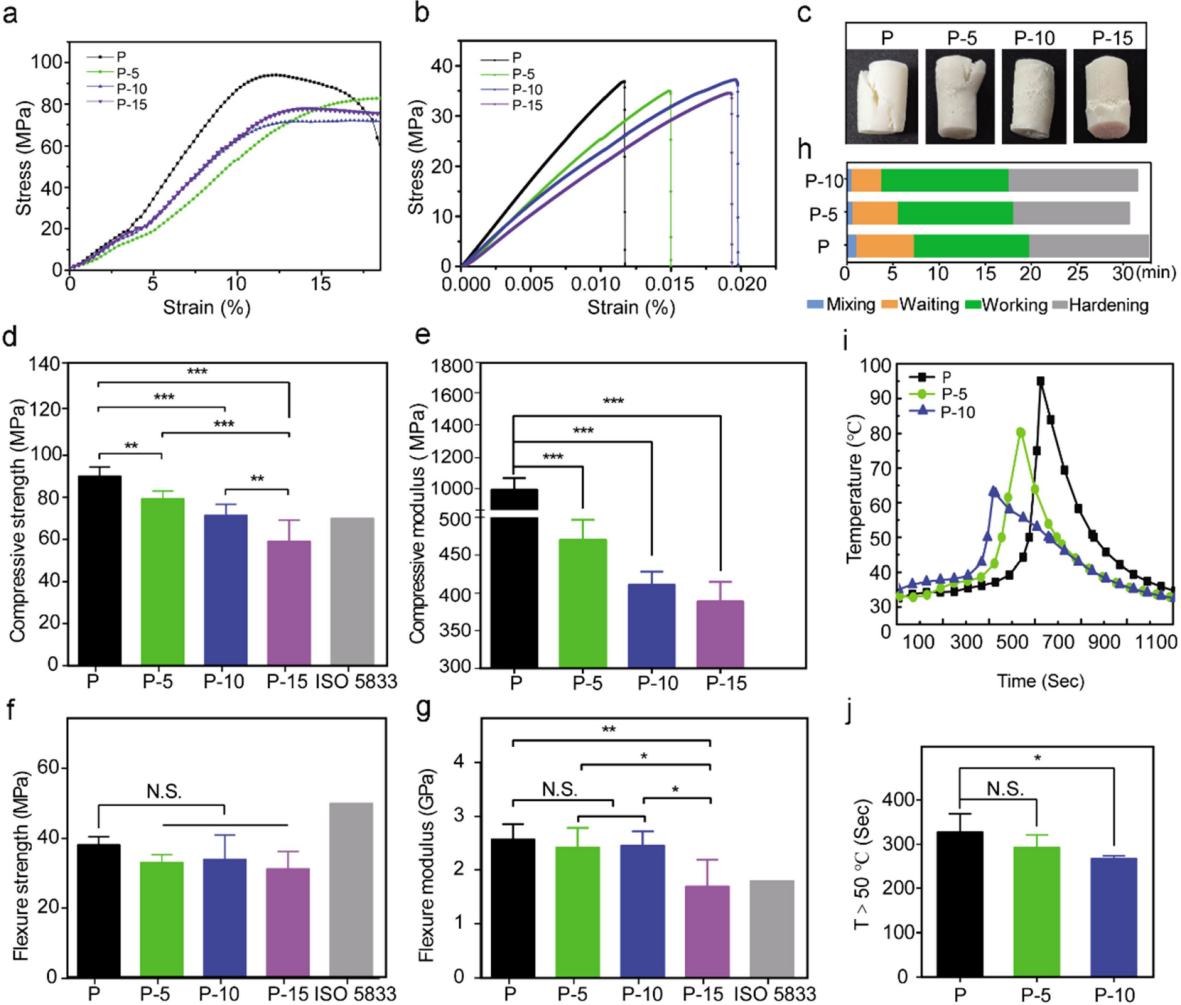
Characterization of the physical properties of SIS-PMMA and PMMA bone cements. The mechanical properties of SIS-PMMA and PMMA bone cement and specifications in ISO5833: representative stress–strain curves after (a) uniaxial compression and (b) three-point flexural tests. (c) Gross views after uniaxial compression tests of each bone cement cylinder. (d) Compressive strength. (e) Compressive modulus. (f) Flexural strength. (g) Flexural modulus. (h) Handing times of bone cement with varying SIS composition. (i) Representative time–temperature curves. (j) Duration of temperature set above 50 °C. *, *p* < 0.05, **, *p* < 0.01, and ***, *p* < 0.001. N.S., not significant. The error bars indicate standard deviations.

The bone cement’s mechanical properties are the most essential factors for vertebral defect repair. Here, the compressive and flexural properties of the SIS-PMMA bone cement were investigated. Figure 3a,b shows the stress–strain curves of the PMMA and SIS-PMMA bone cements. Interestingly, visual inspection after the compression tests showed that PMMA samples were severely deformed with completely crumbled hardened cement clumps. Although the composite P-5 and P-15 bone cement samples only indicated cracks, the P-10 composite bone cement clumps hardly crumbled during axial compression until they fractured (Figure 3c). Furthermore, the compressive modulus was assessed via the stress–strain curve slope in the linear elastic region, which revealed that the compressive strength and modulus were inversely linked to the amount of SIS powder used. Although increased SIS levels reduced the mechanical properties, the compressive strengths of the P-5 and P-10 cements still reached 79.40 ± 3.65 MPa (*p* < 0.01) and 71.48 ± 5.19 MPa (*p* < 0.001), respectively, which were in agreement with the ISO5833 standard (not <70 MPa) and thus suitable for vertebral repair (Figure 3d). The P-15 composite cement compressive strength was 59.01 ± 10.21. The pure PMMA cement indicated the highest compressive modulus (991.48 ± 79.44 MPa), while that of the P-5, P-10, and P-15 composite bone cement dropped to 470.26 ± 26.67 MPa (*p* < 0.001), 411.26 ± 17.11 MPa (*p* < 0.001), and 388.78 ± 25.82 MPa (*p* < 0.001), respectively (Figure 3e), conforming to the range for human trabecular bone (100–700 MPa).

In addition, no notable difference was identified in the flexural strength between PMMA and the three SIS-PMMA bone cement (∼40 MPa) and revealed similar brittle failure behavior (Figure 3b,f). According to the stress–strain curves (Figure 3b), PMMA failed in a brittle manner at low strain values (∼0.012%), while P-5 composite bone cement failed at higher strain values (∼0.015%). With increased SIS levels, the P-10 and P-15 composite bone cement indicated failure at even higher strain values (∼0.02%), suggesting that they had better toughness than PMMA. Moreover, the P-5 and P-10 composites hardly affected the flexural modulus compared with PMMA (Figure 3g). Furthermore, the modulus notably reduced as SIS levels increased up to 15 wt %, where a value of approximately 1.70 GPa ± 0.49 GPa (*p* < 0.01) was obtained.

### 3.4 Handing Times

The handing time for each bone cement, before and after the modification, is illustrated in Figure 3h. The same surgeon repeated the experiment four times, and no substantial difference was observed in total handling time between PMMA and two SIS-PMMA composites (P-5 and P-10). The average mixing time was 57 ± 4 s for PMMA, 33 ± 1 s for P-5, and 27 ± 2 s for P-10. The average waiting time of PMMA, P-5, and P-10 were 6.26 ± 0.34, 5.33 ± 0.2, and 3.2 ± 0.24 min, respectively, whereas the average working time was 12.50 ± 0.2, 12.49 ± 1.05, and 14.22 ± 0.53 min, respectively. The average hardening time for PMMA, P-5, and P-10 was approximately the same, 13.05 ± 0.42, 13.12 ± 1, and 14.11 ± 0.45 min, respectively. According to statistical analysis, SIS addition in PMMA bone cement primarily decreased (*p* < 0.001) in the two stages of the curing process (mixing and waiting) and had no significant difference in the working and hardening time at each mixing weight ratio.

### 3.5 Measurement of Temperature during Polymerization

Figure 3i illustrates the temperature profiles of PMMA and the composite cement samples. The peak temperatures were 84.87 ± 7.56 °C for P-5, 68 ± 6.93 °C for P-10, and 92.17 ± 11.12 °C for PMMA. Moreover, the times above 50 °C were 293 ± 28, 267.25 ± 6.34, and 327.89 ± 41.10 s, respectively. The P-10 composite cement had a markedly reduced time above 50 °C than the PMMA group (Figure 3j). Overall, the time above 50 °C of the cement containing SIS powders decreased and the peak temperature decreased with increasing SIS levels. These data indicated that the SIS in the SIS-PMMA composite cement partially absorbs the heat produced by the exothermic polymerization reaction, thereby decreasing the polymerization temperature and potentially reducing tissue damage when implanted.

### 3.6 DXA Analysis

**Figure 4:**
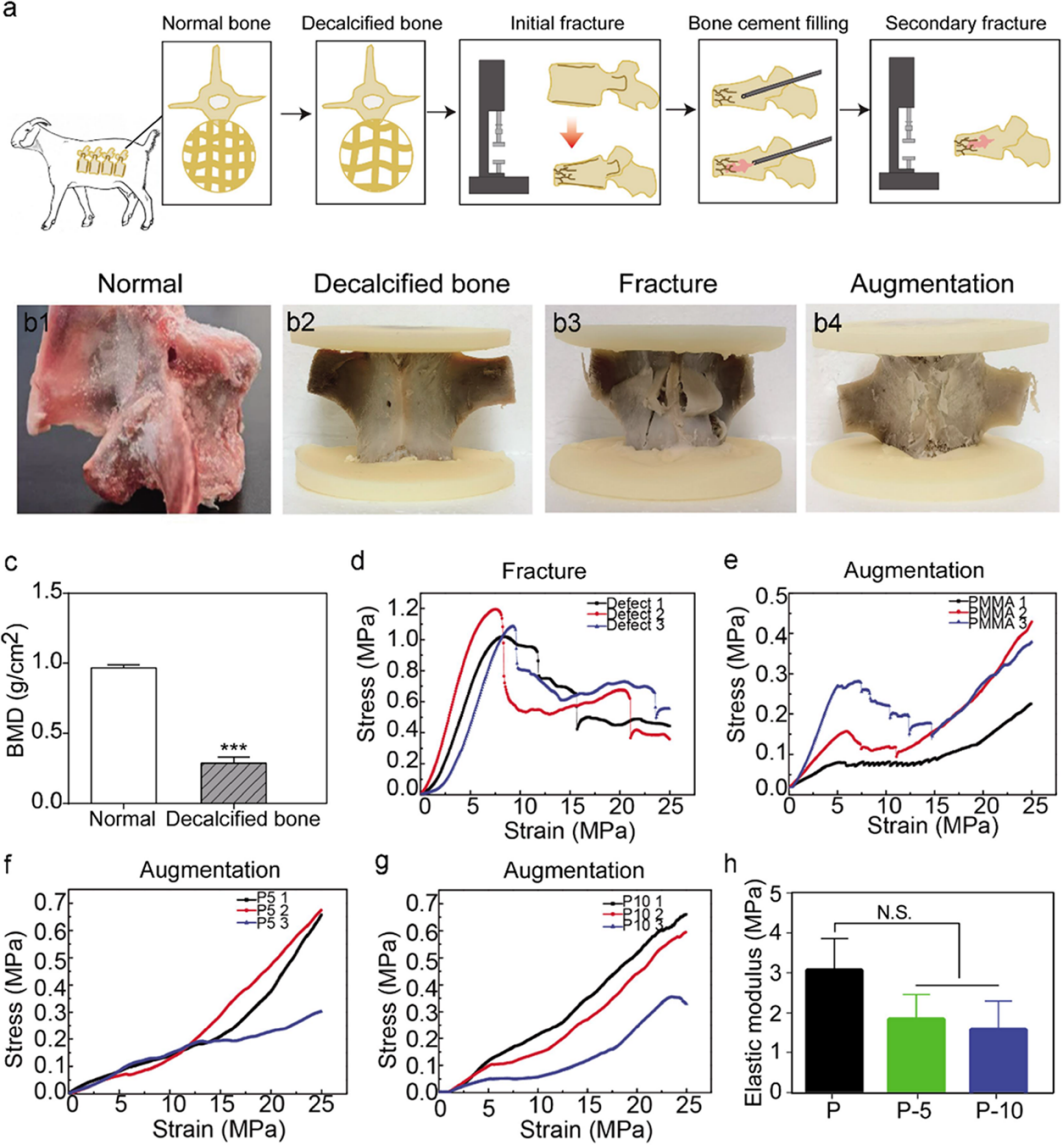
Biomechanical tests in the goat vertebral model. (a) Schematic flowchart of the compression test carried out on the decalcified goat vertebrae augmented by SIS-PMMA and PMMA bone cement. (b) Normal vertebra and decalcified vertebra before simulated compression fracture, with simulated compression fracture, and after cement augmentation. (c) BMD in normal and decalcified vertebra. (d) Initial fracture. Typical mechanical response of decalcified vertebrae after cement augmentation: (e–g) set of stress–strain curves after uniaxial compression (h) and elastic modulus. ***, *p* < 0.001. The error bars represent standard deviations.

In this research, an ex vivo biomechanical evaluation was carried out using decalcified goat vertebral models (Figure 4a). First, the successful osteoporosis model development was confirmed by DXA scanning of pre- and postdecalcification (Figure 4b1,b2). Subsequently, vertebral compression fracture and bone cement augmentation were also performed in vertebrae with reduced BMD (Figure 4b3,b4). As Figure 4c illustrates, in the normal lumbar vertebra, the BMD was 0.968 ± 0.022 g/cm^2^, while in the decalcified lumbar vertebra, it was 0.287 ± 0.044 g/cm^2^, suggesting a significant decrease compared with that of the normal lumbar vertebra. According to the literature, osteoporosis is identified when the spinal bone density is <0.75 g/cm^2^, confirming that the decalcified goat vertebral bodies were osteoporotic in this investigation.

### 3.7 Ex Vivo Biomechanical Evaluation

The strain–stress curve of the defect group (Figure 4d) indicated successful modeling of fracture vertebrae, which were randomly implanted with PMMA (P) and PMMA composed of 5 wt % SIS (P-5) or 10 wt % SIS (P-10). “P, P-5, P-10” represents the secondary fracture of fractured vertebrae that were implanted with PMMA and PMMA composed of 5 wt % SIS (P-5) or 10 wt % SIS (P-10). The observed peak strength of the PMMA-filled vertebrae was 0.157 ± 0.098 MPa. Furthermore, the P-5 and P-10 groups indicated markedly higher compressive strength than the PMMA group (Figure 4e–g). The elastic moduli of vertebral bodies were 1.83 ± 0.62 MPa for the P-5 composite group and 1.59 ± 0.69 MPa for the P-10 composite group, which were not significantly different from that of the PMMA group (3.09 ± 0.76 MPa) (Figure 4h). These results demonstrate that P-5 and P-10 bone cement were biomechanically robust in reducing the secondary vertebrae fracture.

### 3.8 In Vivo Biosafety

**Figure 5:**
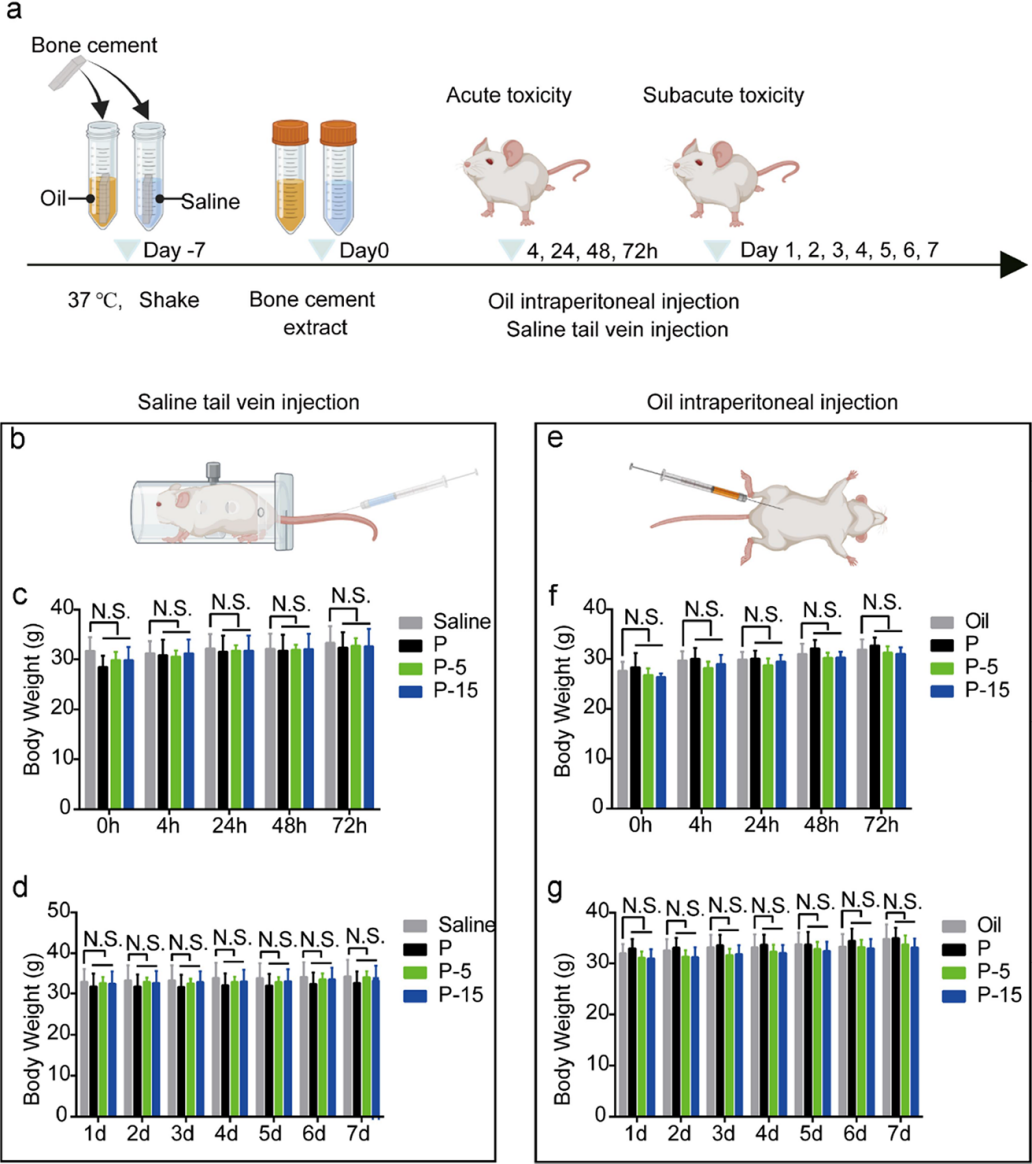
In vivo biosafety evaluation of the SIS-PMMA and PMMA bone cements. (a) Schematic diagram of bone cement extracts preparation and timeline of the in vivo study. Sterile bone cement specimens were incubated with saline and oil for 1 week with a specimen surface area/soaking solution ratio of 6 cm^2^/mL at 37 °C. The polar substances in bone cement were leached out by saline, while the nonpolar substances were leached out by oil. Body weight of SIS-PMMA and PMMA mice after (b) tail vein injection of saline at different time points in (c) acute toxicity and (d) subacute toxicity testing. Body weight of SIS-PMMA and PMMA mice after (e) intraperitoneal injection of oil at different time points in (f) acute toxicity and (g) subacute toxicity testing. N.S., not significant. The error bars represent standard deviations.

Biosafety is a prerequisite for bone substitute materials in vivo. Here, acute/subacute systemic toxicity was comprehensively evaluated. Figure 5a shows the timeline of experimental extract preparation and in vivo treatment. All the mice were alive and healthy, and indicated no toxic reactions, such as prostration, convulsion, or death, during the acute and subacute toxicity experimental period. Furthermore, no marked alterations were observed in the development and body weight of polar (intravenous extract injection shown in Figure 5b) and negative control (intravenous saline injection) groups (Figure 5c,d). Additionally, the mice from the nonpolar groups (intraperitoneal extracts injection) and negative control (intraperitoneal oil injection) groups (Figure 5e) had similar changes in body weight at any selected time points (Figure 5f,g).

For subacute systemic toxicity assessment, at the end of the experiment, routine blood and blood biochemistry analyses were carried out to verify the potential circulatory system or organ damage after polar or nonpolar extract injections. Increase BUN (*p* < 0.05) and CREA level (*p* < 0.05) levels were identified in the P-15/saline and P-5/oil groups, respectively, compared with the negative control groups. There was also an increase in ALT (*p* < 0.05) and CREA (*p* < 0.05) in the P-15/oil and P-5/oil groups, respectively, than the PMMA group; however, these values were within the baseline range and had no clinical significance. Furthermore, all other routine blood parameters (Table 2) and blood biochemistry indexes (Table 3) indicated no significant variability between the experimental and negative control groups (or the PMMA group). Altogether, these data conclude that the composite cement with 5–15 wt % SIS did not elicit acute and subacute systemic toxicity and exhibited good biosafety.

**Table 2:** Hematological Testing of Subacute Systemic Toxicity in a Mouse Model

	saline				oil			
	negative control	PMMA	P-5	P-15	negative control	PMMA	P-5	P-15
WBC (×10^9^/L)	4.69 ± 0.65	4.37 ± 1.63	4.1 ± 0.81	4.07 ± 0.82	9.82 ± 2.01	9.96 ± 2.24	10.54 ± 4.15	10.11 ± 1.02
NE (×10^9^/L)	0.7 ± 0.24	0.62 ± 0.26	0.67 ± 0.2	0.55 ± 0.19	3.23 ± 1.55	3.03 ± 1.17	2.86 ± 2.04	2.75 ± 0.84
MO (×10^9^/L)	0.02 ± 0.04	0 ± 0	0 ± 0	0.03 ± 0.05	0.23 ± 0.15	0.47 ± 0.52	1.42 ± 1.58	0.68 ± 0.57
LY (×10^9^/L)	3.93 ± 0.55	3.72 ± 1.48	3.38 ± 0.73	3.42 ± 0.68	6.33 ± 0.80	6.25 ± 0.94	6.2 ± 2.87	6.55 ± 1.41
EO (×10^9^/L)	0.03 ± 0.02	0.02 ± 0.02	0.03 ± 0.02	0.02 ± 0.02	0.09 ± 0.09	0.19 ± 0.14	0.08 ± 0.07	0.11 ± 0.07
BA (×10^9^/L)	0.005 ± 0.005	0.003 ± 0.005	0 ± 0	0.007 ± 0.008	0.008 ± 0.005	0.02 ± 0.01	0.02 ± 0.01	0.03 ± 0.03
NE (%)	15.2 ± 3.84	14.27 ± 4.88	16.27 ± 4.49	13.7 ± 2.62	31.55 ± 9.62	29.63 ± 5.96	29.74 ± 16.88	27.1 ± 6.6
MO (%)	0.72 ± 0.33	0.57 ± 0.28	0.67 ± 0.3	1.46 ± 1.15	2.03 ± 1.27	4.48 ± 4.77	13.78 ± 12.04	7.1 ± 6.28
LY (%)	83.38 ± 3.78	84.53 ± 5.42	82.48 ± 4.57	84.03 ± 3.59	65.33 ± 10.33	63.92 ± 6.39	55.42 ± 11.13	64.28 ± 8.74
EO (%)	0.6 ± 0.46	0.57 ± 0.62	0.58 ± 0.39	0.63 ± 0.45	1.03 ± 1.32	1.82 ± 1.18	0.86 ± 0.68	1.18 ± 0.85
BA (%)	0.1 ± 0.11	0.07 ± 0.12	0 ± 0	0.18 ± 0.21	0.08 ± 0.05	0.15 ± 0.05	0.2 ± 0.07	0.35 ± 0.33
RBC (×10^12^/L)	9.45 ± 0.44	9.35 ± 0.43	9.53 ± 0.45	9.04 ± 0.60	8.74 ± 1.05	9.25 ± 0.57	9.46 ± 0.50	9.08 ± 0.64
PLT (×10^9^/L)	1374.17 ± 154.35	1355 ± 190.4	1431.5 ± 180.32	1202.67 ± 243.78	1296 ± 166.15	1368.33 ± 193.51	1436.2 ± 262.58	1161.5 ± 228.50
Hb (g/L)	149 ± 4.56	149 ± 10.2	154.5 ± 8.41	145.33 ± 9.79	141 ± 16.02	149.17 ± 7.14	151.2 ± 8.87	148.75 ± 8.54
MCHC (g/L)	287 ± 3.22	288.17 ± 5.04	284.5 ± 5.96	282.5 ± 3.08	282 ± 8.29	290.17 ± 4.45	286.6 ± 7.16	285 ± 2.45
MCH (pg)	15.78 ± 0.52	15.92 ± 0.41	16.22 ± 0.58	16.08 ± 0.2	16.15 ± 0.33	16.12 ± 0.39	16 ± 0.78	16.38 ± 0.26
MCV (fL)	55.05 ± 1.74	55.25 ± 0.73	57.03 ± 1.68	56.87 ± 0.51	57.25 ± 1.87	55.67 ± 1.96	55.84 ± 2.33	57.58 ± 0.47
HCT (%)	51.93 ± 1.58	51.68 ± 3.05	54.33 ± 2.95	51.43 ± 3.12	49.9 ± 4.72	51.42 ± 2.18	52.8 ± 3.12	52.25 ± 3.36
RDW (%)	17.83 ± 0.88	17.95 ± 0.54	17.52 ± 0.53	17.25 ± 1.03	17.63 ± 1.18	18.33 ± 0.90	18.26 ± 0.29	18 ± 0.59

**Table 3:** Biochemical Testing of Subacute Systemic Toxicity in a Mouse Model

	saline				oil			
	negative control	PMMA	P-5	P-15	negative control	PMMA	P-5	P-15
ALT (U/L)	35.17 ± 10.37	41.25 ± 17.55	32.45 ± 7.63	35.75 ± 5.73	26.33 ± 3.79	24.33 ± 4.32	29 ± 6.38	66 ± 36.59^a^
AST (U/L)	147.6 ± 117.43	116.13 ± 64.31	108.37 ± 38.88	115.27 ± 19.59	117.33 ± 21.55	99.17 ± 20.61	164.5 ± 129.07	285.67 ± 148.29
ALP (U/L)	99.57 ± 17.91	82.13 ± 12.96	94.57 ± 22.56	117.89 ± 29.61	77.33 ± 15.01	74.5 ± 9.87	69.75 ± 14.01	78.33 ± 17.62
DBIL (μmol/L)	0.48 ± 0.21	0.51 ± 0.28	0.782 ± 0.38	0.77 ± 0.06	0.42 ± 0.16	0.84 ± 0.99	1.00 ± 0.48	3.15 ± 2.03
TP (g/L)	46.18 ± 1.23	48.1 ± 2.30	48.52 ± 4.81	46.4 ± 1.73	45.33 ± 4.41	45.32 ± 3.6	46.13 ± 2.79	47 ± 1.68
ALB (g/L)	24.8 ± 1.00	26.52 ± 0.99	26.53 ± 2.48	25.13 ± 1.07	24.83 ± 2.24	24.6 ± 1.53	24.85 ± 1.43	25.3 ± 0.82
CA (mmol/L)	2.16 ± 0.09	2.18 ± 0.06	2.21 ± 0.11	2.15 ± 0.06	2.19 ± 0.12	2.18 ± 0.11	2.20 ± 0.03	2.11 ± 0.03
BUN (mmol/L)	9.38 ± 1.32	10.12 ± 0.82	10.58 ± 1.14	11.3 ± 0.72^b^	6.63 ± 0.67	6.48 ± 0.55	7.8 ± 1.68	7.43 ± 1.16
CREA (μmol/L)	13.05 ± 2.24	12.73 ± 2.36	14.08 ± 2.75	14.93 ± 1.26	11.67 ± 0.58	12.17 ± 0.98	15.25 ± 2.22^a^^,^^b^	13.33 ± 1.15
GLU (mmol/L)	15.03 ± 3.75	13.02 ± 3.48	13.92 ± 4.04	14.93 ± 2.24	12.84 ± 3.56	13.62 ± 3.39	13.45 ± 2.02	12.13 ± 2.66
K (mmol/L)	5.53 ± 0.67	5.61 ± 0.59	5.28 ± 0.42	5.18 ± 0.22	5.37 ± 0.67	5.04 ± 0.20	6.07 ± 1.30	5.47 ± 0.96
Na (mmol/L)	146.53 ± 1.95	146.99 ± 1.62	151.145 ± 5.05	148.685 ± 0.5	145.03 ± 1.64	145.18 ± 1.59	146.18 ± 0.68	144.47 ± 2.303
Cl (mmol/L)	108.46 ± 2.79	109.36 ± 1.87	112.135 ± 2.38	109.12 ± 1.51	108.73 ± 1.54	108.37 ± 1.4	108.5 ± 1.57	107.33 ± 0.896

a Values are significantly different from those of the PMMA cement (#, *p* < 0.05).

b Values are significantly different from those of the negative control (*, *p* < 0.05).

**Figure 6:**
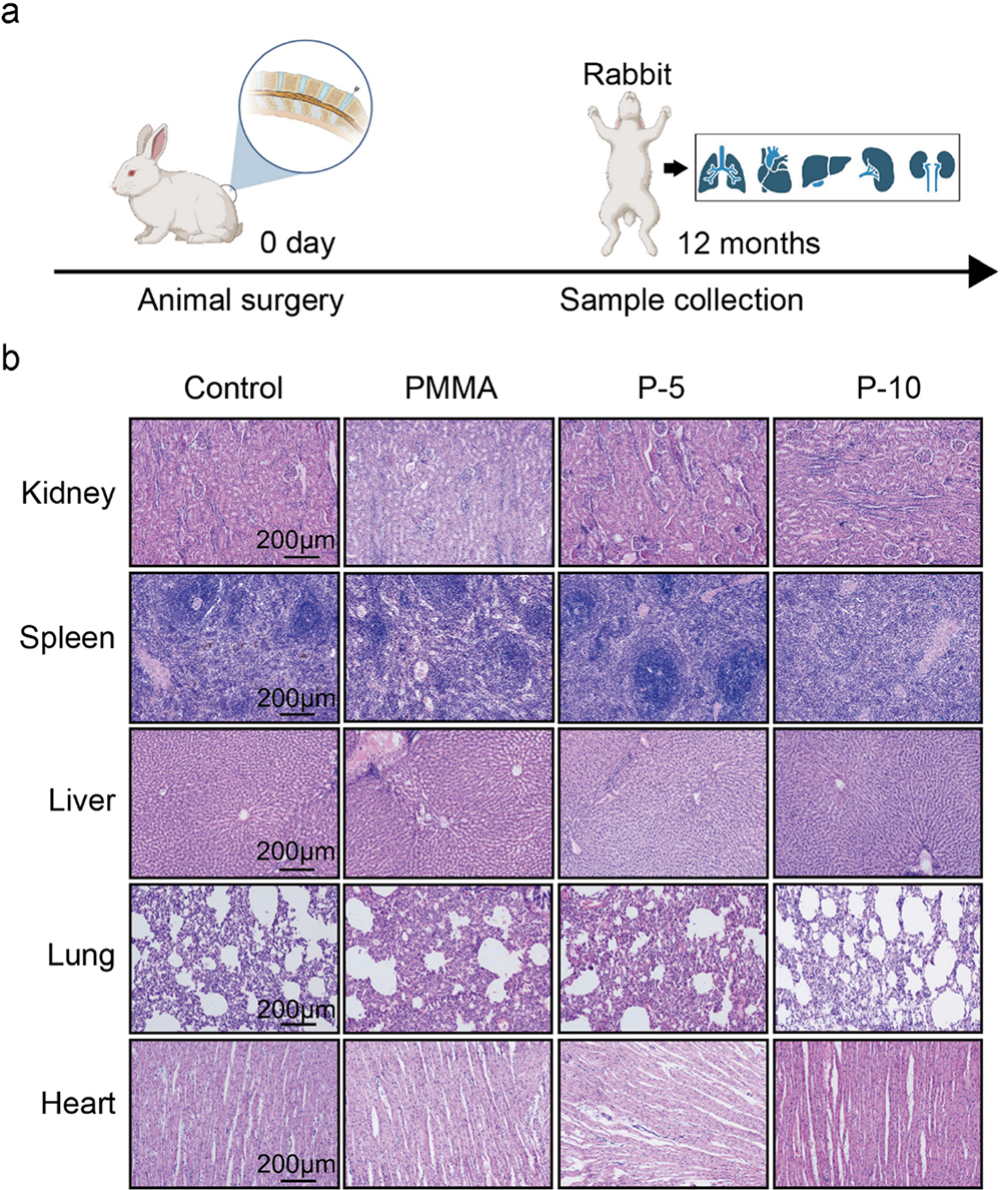
Organ toxicity of SIS-PMMA and PMMA implantation in a rabbit vertebral defect model. (a) Schematic diagram illustrating the chronic toxicity test in the viscera, including the liver, lung, spleen, heart, and kidney of the rabbit model. (b) HE staining of rabbit organs harvested 12 weeks postsurgery indicated no obvious morphological differences in the heart, lungs, kidneys, liver, or spleen among different groups. For each group, 4 rabbits were assessed, where at least 10 sections were observed at different sites for each rabbit. Scale bar = 200 μm.

As shown in Figure 6, the chronic toxicities of the spleen, lung, heart, liver, and kidney after composite bone cement augmentation were also evaluated. According to the histological analysis, there were no noticeable pathological alterations, proving that the composite 5 and 10% SIS cement were nonallergenic and biocompatible. The major reason for the safety of SIS-PMMA composite cements might be that their components are all safe for in vivo bone substitution. FDA has authorized the clinical use of SIS. PMMA bone cement has been frequently used as a bone defect filler, repair material, and grouting agent in vertebroplasty for >30 years. Therefore, using SIS-PMMA as a bone repair material is notably biocompatible, clinically acceptable, and beneficial for OVCF patients.

### 3.9 Rabbit Vertebral Defect Model

**Figure 7:**
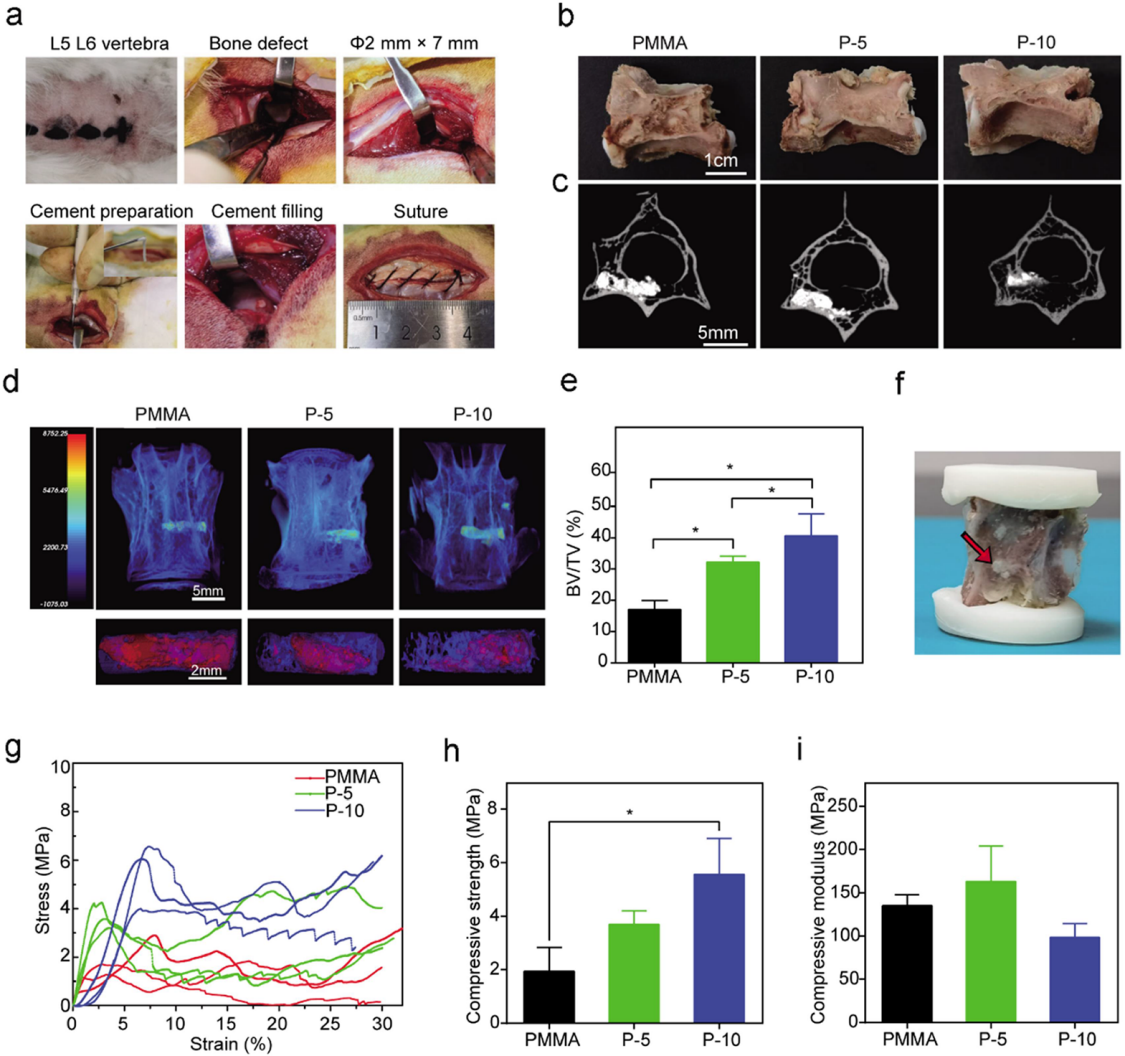
In vivo bone ingrowth and biomechanical evaluation in the rabbit model. (a) Surgical procedure of PMMA and SIS-PMMA bone cement implantation in rabbit vertebral defects. (b) Representative optical photographs of vertebrae implanted with PMMA, P-5, and P-10 bone cement after 12 weeks. (c) Micro-CT scanning of the transverse sections of vertebra samples. (d) 3D-reconstructed images of the vertebrae with different CT values are represented by different colors (top); blue indicates the new bone, and red indicates bone cement in each group (bottom). (e) Statistical assessment of new bone and tissue volumes (BV/TV). Eight vertebra samples were measured for each group. (f) Experimental vertebral bodies with cement were extracted 12 weeks after implantation, and a representative photograph was taken before the compression assay. (g) Stress–strain curves of vertebrae filled with cement. Three vertebra samples were measured for each group. (h) Compressive strength and (i) compressive modulus of the defective vertebrae and those filled with different types of cement.

For an in vivo evaluation of the bone cements, rabbit vertebral defect models were established. With the help of a 14G bone marrow cannula, the PMMA and two SIS-PMMA bone cement were injected into the L5 and L6 vertebrae of rabbits surgically (Figure 7a). After 12 weeks of bone cement injections, gross observation displayed holes that were closely interconnected with PMMA and two SIS-containing composites (Figure 7b). The transverse section of micro-CT images showed two SIS-containing groups, especially the P-10 composite bone cement, which degraded with apparent remodeling of lacunae and bone structure replacement (Figure 7c); however, the PMMA group indicated almost no degradation and had the largest bone cement volume compared with the P-5 and P-10 composites.

The 3D rendering by micro-CT indicated the location of the defect and bone cement (Figure 7d). The bone cement had a high CT value and was shown in red and yellow, while the bone was shown in blue. The color alters to green and finally to blue as the bone cement is replaced with bone, suggesting gradually lowering CT values. At 12 weeks, the PMMA group had the highest CT value and decreased as the SIS level increased in the composite groups. Compared with PMMA and P-5, in the P-10 composite group, the areas with CT values were more similar to those of bone. In Figure 7d, the 3D-reconstructed images on the bottom show each group’s volume of new bone (blue) and bone cement (red) in the defect. In the P-5 and P-10 groups, there was an obvious new trabecular bone in the defect, while only a few bone formations were identified in the PMMA defects. These findings revealed that there was more material resorption and bone growth in the SIS-PMMA groups than in the PMMA group. At 12 weeks postsurgery, the quantitation of the reconstructed 3D images (*n* = 8) of the vertebral body indicated higher bone formation in the SIS-PMMA groups than in the PMMA group (Figure 7e). Statistically, there was a high percentage of new bone volume (BV/TV, 40.65 ± 6.93%, *p* < 0.05) in the P-10 than in the P-5 (BV/TV, 32.22 ± 1.85%, *p* < 0.05) and PMMA groups (BV/TV, 17 ± 3%). Moreover, increased bone growth was identified in composite groups with a higher SIS proportion.

### 3.10 Compressive Properties

Figure 7f represents the bone cement position in the rabbit vertebral augmentation model for the compression test. The compressive strength was defined as when the stress reached a failure point. Three vertebra samples were measured for each group. Regarding the stress–strain curve images (Figure 7g), P-10 showed a higher peak strength than PMMA and P-5. The compressive strength of the PMMA group was 1.94 ± 0.89 MPa, which was lower than P-5 (3.69 ± 0.51) and P-10 (5.56 ± 1.35, *p* < 0.05) (Figure 7h). Using the slope of the stress–strain curve in the linear elastic region, the compressive modulus was assessed. The compressive modulus of PMMA was 135.27 ± 12.32 MPa, while that of 5 and 10 wt % SIS-PMMA bone cement was 163.25 ± 40.77 and 98.54 ± 15.75 MPa, respectively, which were not significantly different from that of the PMMA cement (Figure 7i).

### 3.11 Histological Analysis

**Figure 8:**
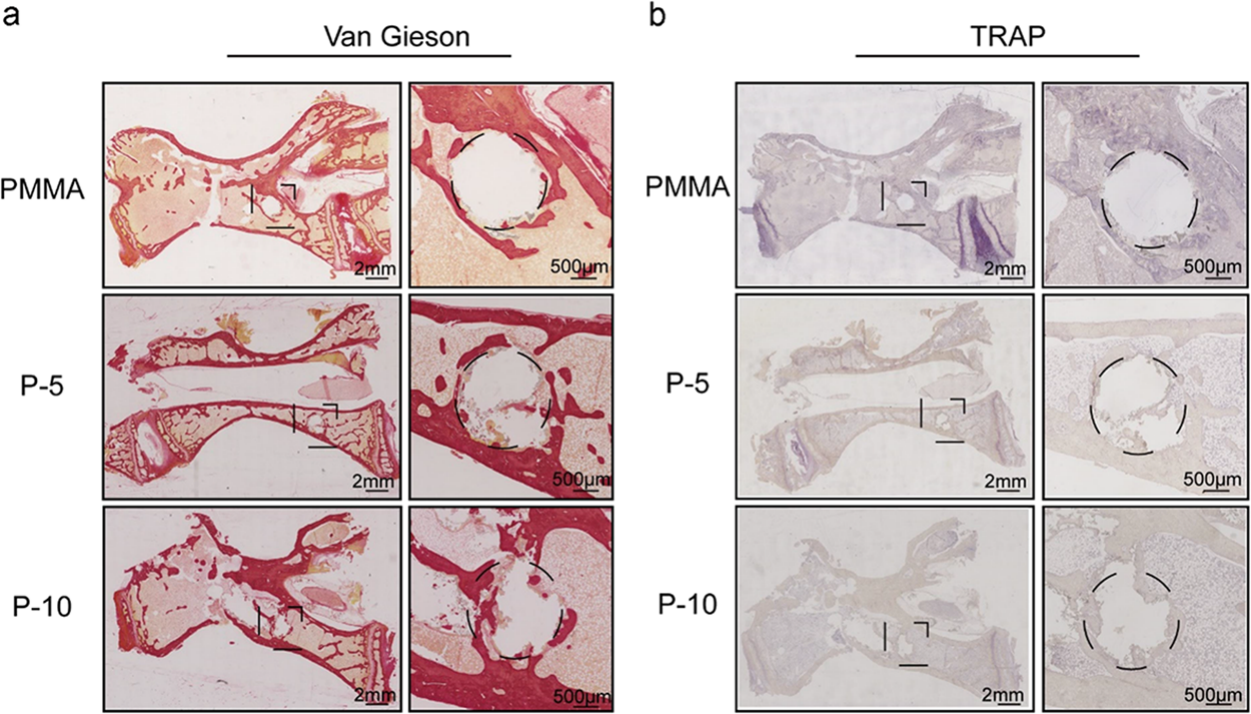
Histological staining of hard tissue sections from vertebral defects implanted with PMMA and SIS-PMMA bone cement at 12 weeks. Representative images of (a) Van Gieson staining and (b) TRAP staining near the defects. The corresponding square boxes represent higher-magnification images. New bone tissue around the bone–cement interface was identified in all SIS-PMMA groups, where the P-10 group revealed the most developed structure. However, the PMMA group showed poor bonding with the host bone. For each group, 5 vertebra samples were assessed, and in each rabbit, at least 6 sections from different sites were measured.

After 12 weeks of cement injection, histological analysis was carried out on the acquired samples. The treated vertebra hard tissue sections were stained with fuchsin and picric acids to identify bone repair (Figure 8a). Only a few inflammatory cells were identified in all of the groups. Deep red areas indicate bone tissue, while light red regions indicate osteoblasts and bone marrow. The SIS-PMMA groups (P-5 and P-10) indicated significant bone ingrowth as well as new bone and bone marrow formation. However, no regeneration was identified in the PMMA group since the boundary of the bone cement was intact and smooth, with only slight bone repair. In addition, few TRAP-positive osteoclasts were observed in the two composite and PMMA bone cement groups (Figure 8b).

## 4 Discussion

Currently, PMMA cement is the frequently employed injectable filler material in orthopedic surgery, especially for PVP and PKP.^2,3^ However, PMMA has certain shortcomings, such as high polymerization temperature, high elastic modulus, and poor osteointegration, which increases the postsurgical risk of side effects.^29,30^ Here, the natural biomaterial SIS, which has good biocompatibility and osteogenic activity, was utilized for modifying PMMA bone cement. Furthermore, the optimal ratio and SIS powder size for the SIS-PMMA cement were determined. Moreover, in vivo and in vitro analyses were conducted to prove that SIS modification improves biocompatibility, polymerization temperature, mechanical properties, handling time, biosafety, and osteogenesis of PMMA cement. Specifically, the in vivo assays provide evidence of the effectiveness and safety of SIS-PMMA bone cement, which is crucial to its clinical application.

The PMMA bone cement’s effectiveness primarily depends on its mechanical properties.^31^ Some literature has correlated bone cement modulus with the risk of vertebral refracture after vertebroplasty.^32,33^ Pure PMMA bone cement has a significantly higher elastic modulus (*E*) than that of the surrounding vertebral cancellous bone (*E*_cement_ = 2000–3700 MPa; *E*_bone_ = 100–700 MPa).^13,34^ Furthermore, researchers have tried to reduce the compressive modulus of PMMA bone cement, for example, by adjusting the monomer component or by adding additives (such as calcium phosphate cement, polymer, etc.) in the powder part.^35,36^ Some researchers used composite cement to reduce the PMMA compressive strength; however, it did not meet the requirement of ISO 5833.^37−39^ In this research, SIS-PMMA bone cement (5, 10, and 15 wt % SIS) indicated reduced elastic modulus compared to pure PMMA bone cement, achieving mechanical properties similar to those of host bone. In addition, the compressive strengths of both the P-5 and the P-10 composite bone cement satisfied the standing acrylic bone cement requirement of >70 MPa. Therefore, the SIS-PMMA hybrid cement might meet the clinical standard for mechanical strength for treating OVCFs with PKP and PVP.

Currently, most literature mainly focuses on the mechanical characteristics of modified bone cement^27,40^ and lacks the biomechanical clinical outcomes to compare the biomechanical advantages of modified PMMA over traditional PMMA during vertebroplasty. In this research, a simulated osteoporotic goat vertebral compression fracture model and rabbit vertebral defect model were utilized for assessing the biomechanical stability of the SIS-PMMA cement. The data indicated that vertebral bodies with SIS-PMMA bone cement (P-5, P-10) augmentation have a substantially increased maximum strength compared to those with only PMMA cement, which might be partly because SIS powder enhanced toughness. In addition, SIS has osteoinductive properties, beneficial for trabecular bone regeneration. New bone is formed and connected to the SIS by bone conduction, which enhances the vertebral body strength as a whole.

One of the biological concerns in the clinical application of SIS-PMMA bone cement is its toxicity. Theoretically, unpolymerized monomers and residual radicals after PMMA cement polymerization can lead to biological toxicity.^40^ In the present research, SIS (>90% is collagen) was incorporated through physical bonding in the PMMA polymer powder. In order to ensure the most complete polymerization and minimize monomer toxic effects, the volume of MMA is used for the SIS-PMMA groups, as per the manufacturer’s guide for commercial PMMA. Following the international standard ISO 16886-11, the in vivo study indicated that SIS-PMMA had satisfactory biosafety regarding acute and subacute systemic toxicity and chronic lung, liver, kidney, heart, and spleen toxicity. Previously, it has been indicated that monomer MMA and additives released from unpolymerized resinous materials may diffuse in the host bone tissues and trigger an adaptive response in the surrounding cells.^41,42^ Here, the in vitro Transwell insert model was utilized to mimic this clinical situation, which revealed that the incorporation of SIS into PMMA had no obvious cytotoxicity and even promoted cell proliferation. Additionally, during polymerization, there was no significant difference in total handling times between PMMA and SIS-PMMA (for both P-5 and P-10). Therefore, it was inferred that the inclusion of a specific proportion of SIS may have little to no impact on the process of PMMA polymerization.

Moreover, for preclinical biocompatibility for long-term bone implantation, SIS-PMMA was studied in vivo in rabbits to evaluate the surface bonding. The micro-CT data indicated no bone cement leakage or implantation failure in the experimental vertebrae. Furthermore, the volume of regenerated bone around the SIS-PMMA was more than that around the PMMA. Additionally, the histological assessment indicated that bone repair was better after SIS-PMMA bone cement was filled in the bone defects. This increased osseointegration could be linked with SIS as PMMA is biologically inert and furnishes mechanical support only for fractured vertebral bodies but cannot biodegrade and promote fracture union. According to Chiu et al., PMMA bone cement not only inhibits transcription factors that accelerate osteoprogenitor differentiation but also suppresses osteoprogenitor viability.^43,44^ Our previous studies suggested that SIS can be degraded and replaced by host tissue after implantation in several animal bone defect models.^17,23−25,45^ As SIS degrades, a pore structure is formed, and the released growth factors, such as transforming growth factor-β (TGF-β), basic fibroblast growth factor (bFGF), and vascular endothelial growth factor (VEGF), promote expansion and osteoblast proliferation.

The ideal bone cement for fractured vertebra treatment should possess an appropriate microstructure and sufficient porosity.^46,47^ Here, the SEM images indicated obvious pore formation in the SIS-PMMA cement with homogeneous dispersion of SIS in PMMA (Figure 1f). Moreover, the cured bone cement pore numbers can be regulated by altering the powder size range and the amounts of the SIS powders added. Additionally, the P-10 composite group (SIS powder passed through a 100-mesh test sieve) had more pores than other groups. The pore formation has been reported to decrease stiffness and increase the osteointegration capability of the material,^48,49^ consistent with the results of this investigation (Figures 1, 3, 8). Moreover, the pore formation in basic materials could also reduce its thermal properties,^48^ in line with this research. The maximum SIS-PMMA composite temperature was decreased compared to pure PMMA cement. The maximum reduction in Tmax was 26.2%, from 92.17 ± 11.12 °C in the PMMA samples to 68 ± 6.93 °C in the P-10 samples (Figure 3i). These values are very promising as they suggest that using SIS-PMMA cements in humans could reduce the risk of thermal injury to neighboring tissues. However, the decreased temperature resulted in the reduced setting time, thereby shortening the handling time from mixing to hardening. Our experiments found that incorporating SIS into PMMA bone cement mainly decreased (*p* < 0.001) the mixing time and waiting time, but not the working time. It has no substantial effect on the surgical process. Jiang^50^ reported that Mendec Spine bone cement had an overlong setting time compared with Spineplex and Osteopal V bone cement during vertebroplasty, which was reduced by approximately 20.0% after modification by MC, making it more convenient for surgical application.

## 5 Conclusions

This research developed a novel SIS-PMMA bone cement by incorporating SIS powder in commercially available PMMA bone cement powder. The cross-sectional SEM images showed that the SIS-PMMA bone cement (SIS powder passed through a 100-mesh test sieve) had advantages in pore numbers compared to those passed through 50- or 200-mesh test sieves. Therefore, the former SIS-PMMA (100-mesh) composite was selected for further study. This investigation indicated that the SIS-PMMA composite partially overcame the drawbacks of traditional PMMA, as it exhibited a more porous structure, substantially reduced stiffness, lower polymerization temperature, and better osteoconductivity and osteoinductivity than traditional PMMA bone cement. Furthermore, in comparison with the commercialized bone cement (Mendec Spine), SIS-PMMA consistently indicated good biosafety and better mechanical response in various standardized animal models. The in vivo micro-CT analysis to quantify the new bone formation ratio and histological analysis of the rabbit vertebral defect model revealed that the osseointegration and bone formation of the SIS-PMMA composites were better than PMMA bone cement. Moreover, the SIS addition of the PMMA bone cement did not have a marked influence on cement processing times. Overall, these data indicate that SIS-modified PMMA bone cement is a potential product for vertebral augmentation in PVP and PKP. However, further research to assess the durability and stability of SIS-PMMA over an extended period and with longer-term follow-up is needed.

## Abbreviations

PMMA: poly(methyl methacrylate)
OVCFs: osteoporotic vertebral compression fractures
SIS: small intestinal submucosa
PVP: percutaneous vertebroplasty
PKP: percutaneous balloon dilatation
HA: hydroxyapatite
β-TCP: β-tricalcium phosphate
ECM: extracellular matrix
BMSCs: bone mesenchymal stem cells
SEM: scanning electron microscopy
FBS: fetal bovine serum
PBS: phosphate buffered saline
αMEM: minimum essential medium α
EDTA: ethylene diamine tetra acetic acid
PTFE: polytetrafluoroethylene
BMD: bone mineral density
WBC: white blood cell
NE: neutrophil
LY: lymphocyte
MO: monocyte
EO: eosinophilic granulocyte
BA: basophilic granulocyte
Hb: hemoglobin
MCH: mean corpuscular hemoglobin
RBC: red blood cell
MCV: mean corpuscular volume
HCT: hematocrit
PLT: platelet
RDW: red cell distribution width
AST: aspartate aminotransferase
ALT: alanine aminotransferase
ALP: alkaline phosphatase
TP: total protein
ALB: albumin
GLU: blood glucose
CREA: creatinine
BUN: blood urea nitrogen
DBIL: direct bilirubin
K: potassium
Na: sodium
Cl: chlorine
Ca: calcium
micro-CT: microcomputed tomography
H&E: hematoxylin-eosin
TRAP: tartrate resistant acid phosphatase
